# Astrocyte subtype‐specific alterations in the dentate gyrus of individuals with mesial temporal lobe epilepsy

**DOI:** 10.1002/epi4.70324

**Published:** 2026-08-01

**Authors:** Chiara Lötzsch, Jakob Johann Schmidt, Daniel Delev, Roland Coras, Ingmar Blümcke, Ruth Beckervordersandforth

**Affiliations:** ^1^ Institute of Biochemistry Friedrich‐Alexander‐Universität Erlangen‐Nürnberg Erlangen Germany; ^2^ Department of Neuropathology Universitätsklinikum Erlangen Erlangen Germany; ^3^ Department of Neurosurgery Universitätsklinikum Erlangen Erlangen Germany

**Keywords:** astrocytes, astrogliosis, dentate gyrus, hippocampal sclerosis, mesial temporal lobe epilepsy

## Abstract

**Objective:**

Epilepsy affects approximately 50 million people worldwide and, although primarily attributed to neuronal dysfunction, increasing evidence highlights a critical role of glial cells, particularly astrocytes, in the pathophysiological mechanisms. Mesial temporal lobe epilepsy (MTLE), the most common form of drug‐resistant epilepsy, is frequently associated with hippocampal sclerosis (HS) and pronounced astrogliosis. Given the limited efficacy of current anti‐seizure medication (ASM) and the side effects of surgical hippocampus removal, there is a need for more specific and effective therapies that potentially address non‐neuronal mechanisms. Astrocytes, with their inherent heterogeneity, are key candidates for such approaches due to their role in regulating and maintaining neuronal activity.

**Methods:**

To investigate subtype‐specific astrocyte alterations in MTLE, we performed immunofluorescence analyses of human dentate gyrus (DG) tissue from MTLE patients (HS1, HS2, and noHS according to the International League Against Epilepsy (ILAE) consensus classification of HS in temporal lobe epilepsy) and postmortem controls. We analyzed the expression and spatial distribution of selected functionally relevant astrocytic proteins, including glial fibrillary acidic protein (GFAP), glutamine synthetase (GS), excitatory amino acid transporter 2 (EAAT2), and aquaporin‐4 (AQP4).

**Results:**

In control tissue, astrocyte subtypes, characterized by combinatorial expression of GFAP, GS, EAAT2, and AQP4, displayed distinct, layer‐specific protein expression profiles across the DG compartments. While the subtype identities were largely preserved in MTLE, localization and expression levels of GFAP, EAAT2, and AQP4 were dramatically altered, suggesting functional deficits in glutamate transport and water homeostasis. Since GS expression was unaffected by MTLE, it served as a proxy to quantify the number of astrocytes. In contrast to existing reports, we found that astrocyte numbers did not differ between control and MTLE patients.

**Significance:**

Our findings demonstrate that astrocyte reactivity in MTLE is not uniform but occurs in a subtype‐ and region‐specific manner. This highlights astrocyte heterogeneity as an important feature of MTLE pathology and underscores the need to consider astrocyte diversity in understanding disease mechanisms and developing precision medicine‐based therapeutic strategies.

**Plain Language Summary:**

Epilepsy is usually studied as a disease of nerve cells, but support cells, called astrocytes, are also involved. We studied brain tissue from patients with a common form of drug‐resistant epilepsy and found that different groups of astrocytes showed distinct changes in proteins that help control brain activity and water movement in the brain. Although the overall number of astrocytes did not change compared with postmortem control patients, the cells displayed strong signs of disease‐related reactivity. Our findings highlight astrocytes as potential targets for future epilepsy treatments.

**Key points**

- Astrocyte subtype organization is preserved across dentate gyrus layers in MTLE.
- Astrocyte reactivity in MTLE follows layer‐ and subtype‐specific patterns rather than a uniform gliotic transformation.
- Reactive astrogliosis is linked to GFAP upregulation without a concomitant increase in astrocyte numbers.
- EAAT2 and AQP4 display a diffuse redistribution pattern in molecular layer astrocytes in MTLE.
- Distinct astrocyte subtype organization in MTLE may provide novel targets for precision epilepsy therapies.

## 1 INTRODUCTION

Epilepsy is a chronic neurological condition characterized by unprovoked, spontaneous seizures resulting from pathological synchronization and hyperexcitability within neuronal networks. Approximately, 50 million individuals^1^ worldwide suffer from focal or generalized epilepsy. MTLE is the most common form of focal epilepsy and represents a leading cause of pharmaco‐resistant focal seizures.^2^, ^3^, ^4^, ^5^ Existing ASMs primarily target neurons, yet approximately one‐third of patients remain resistant to medical treatment.^6^ Moreover, ASMs frequently entail adverse effects, including fatigue, cognitive decline, and concentration disorders.^7^, ^8^ Consequently, surgical resection of the epileptogenic focus remains the most effective treatment option for many patients, particularly those with HS, resulting in freedom from disabling seizures in more than 70% of patients two years postoperatively.^9^ In MTLE, HS is the most common histopathological lesion and is characterized by region‐specific neuronal loss and astrogliosis. According to the ILAE consensus classification of HS in temporal lobe epilepsy, HS can be subdivided into distinct subtypes based on the pattern of neuronal loss and gliosis. HS Type 1 is characterized by severe neuronal loss and gliosis in Cornu Ammonis sector 1 (CA1) and CA4, HS Type 2 by predominant neuronal loss and gliosis in CA1, and HS Type 3 by predominant neuronal loss and gliosis in CA4. In contrast, the noHS group exhibits largely preserved neuronal density with reactive gliosis only, and is associated with poorer epileptological and neuropsychological outcomes after surgery.^10^, ^11^ In the present study, patients were classified into HS1, HS2, and noHS groups according to these established histopathological criteria, based on routine neuropathological assessment of surgical specimens by experienced neuropathologists (Table 1).

**TABLE 1:** Clinical findings of individuals included in the study.

Patient ID	Class	Sex	Age@surgery	Side	Age@onset	Duration	Outcome	ASM pre‐op	ASM post‐op	Relevant medical history, comorbidities
ID1	HS1	F	36	L	18	17	n.a.	CBZ	n.a.	Febrile seizures in the 1st year of life
ID2	HS1	F	25	R	4	21	1b	LEV, LTG	LEV, ZNS, PER	/
ID3	HS1	M	6	L	3	3	n.a.	VPA, OXC, LTG	n.a.	Intracranial hemorrhage due to prematurity
ID4	HS1	M	37	R	0.75	37	4b	VPA, TPM, PRM, LEV	PRM, OXC, BRV	Meningoencephalitis in the 1st year of life
ID5	HS1	F	23	L	2	21	1d	LEV, LTG	/	Febrile seizures in the 2nd year of life
ID6	HS1	F	22	R	2	20	1a	VPA, LEV, OXC	n.a.	/
ID7	HS1	F	41	L	9	32	1b	LEV	LEV, LCM	Meningoencephalitis, FCD IIIa
ID8	HS1	M	34	R	7	28	1b	LEV, VPA, CBZ, OXC	LTG	Febrile seizures at 10 months of age
ID9	HS1	M	42	R	n.a.	n.a.	n.a.	n.a.	n.a.	n.a., FCD IIIa
ID10	HS1	F	60	R	10	50	1a	LEV, CBZ, VPA, OXC, CZP	LEV, CBZ	/
ID11	HS2	F	32	R	28	4	1a	LEV, LTG	/	Suspected febrile seizure in childhood
ID12	HS2	F	29	L	1	28	n.a.	LTG, CBZ	n.a.	Febrile seizure at 9 months of age
ID13	HS2	F	15	L	10	5	n.a.	CBZ, LTG, VPA	n.a.	Febrile seizures at 7 and 17 months of age
ID14	HS2	F	3	R	1	2	n.a.	OXC, TPM	n.a.	Intrauterine infarction
ID15	HS2	M	41	R	2	39	3a	VPA, OXC	OXC, CNB, BRV	/
ID16	HS2	M	15	R	4	11	n.a.	LTG, LEV, VPA, OXC	n.a.	Perinatal asphyxia, glial scar, FCD type IIId
ID17	HS2	F	40	R	4	36	1a	CBZ, VPA, LEV	n.a.	Meningitis in the 3rd year of life, FCD type IIIa
ID18	HS2	F	56	R	8	48	2a	LEV, OXC, LTG, PRM	LEV, OXC, LCM, PRM	Traumatic brain injury
ID19	HS2	F	41	L	4	37	n.a.	LEV, OXC, CLB	n.a.	Febrile seizure at 9 months of age
ID20	HS2	M	4	R	2	2	n.a.	LEV, TPM, OXC, VPA	n.a.	Herpes‐Encephalitis, glial scars
ID21	HS2	M	40	R	16	24	1a	LEV, CBZ, LTG	/	Meningococcal meningitis at 8 months of age
ID22	noHS	M	26	R	24	2	4a	n.a.	n.a.	Encephalitis
ID23	noHS	F	42	L	26	16	1a	LEV, OXC	/	/
ID24	noHS	M	37	L	19	18	4a	PGN, OXC, VPA, CBZ, LEV	LTG, PER	/
ID25	noHS	M	48	R	35	13	1a	LEV, OXC	/	/
ID26	noHS	F	15	L	4	11	n.a.	n.a.	n.a.	n.a., dysembryoplastic neuroepithelial tumor
ID27	noHS	M	10	R	0	10	n.a.	VPA, TPM, LEV, OXC	n.a.	/, FCD type Ia
ID28	noHS	M	20	R	9	11	n.a.	VPA, LEV, LTG, TPM	n.a.	Sturge–Weber syndrome, glial scar/FCD type IIIc
ID29	noHS	M	43	R	25	18	3a	CBZ, LTG, LEV, VPA	LTG, LCM	/, arachnoid cyst
ID30	noHS	F	8	R	0	8	n.a.	LEV, VPA, TPM, CBZ	n.a.	/, FCD type IIb
ID31	noHS	F	3	R	0	3	n.a.	LEV, VPA, LTG, OXC, CBZ	n.a.	Tuberous sclerosis, cortical tuber
ID32	Autopsy control	M	68	L	X	x	X	X	X	Normal hippocampus, cause of death: respiratory failure
ID33	Autopsy control	F	67	R	X	X	X	X	X	Normal hippocampus, cause of death: n.a.
ID34	Autopsy control	F	44	L	X	X	X	X	X	Normal hippocampus, cause of death: hemorrhagic shock
ID35	Autopsy control	F	63	R	X	X	X	X	X	Normal hippocampus, cause of death: cardiac arrest
ID36	Autopsy control	M	54	R	X	X	X	X	X	Normal hippocampus, cause of death: pulmonary embolism
ID37	Autopsy control	M	63	R	X	X	X	X	X	Normal hippocampus, cause of death: cardiac arrest
ID38	Autopsy control	M	58	R	X	X	X	X	X	Normal hippocampus, cause of death: cardiac arrest
ID39	Autopsy control	M	47	R	X	X	X	X	X	Normal hippocampus, cause of death: hemorrhagic shock
ID40	Autopsy control	M	74	L	X	X	X	X	X	Normal hippocampus, cause of death: n.a.
ID41	Autopsy control	M	74	R	x	X	X	X	X	Normal hippocampus, cause of death: cardiopulmonary failure

*Note*: HS classification according to ILAE criteria^10^; Sex: male (M), female (F); Age@surgery: age at epilepsy surgery in years; Side: side of hippocampal resection was right (R) or left (L); Age@onset: age at epilepsy onset in years; Duration: duration of epilepsy in years; Outcome: Postsurgical outcome according to Engel classification.

Abbreviations: BRV, brivaracetam; CBZ, carbamazepine; CLB, clobazam; CNB, cenobamate; CZP, clonazepam; LCM, lacosamide; LEV, levetiracetam; LTG, lamotrigine; n.a, not available; OXC, oxcarbazepine; PER, perampanel; PGN, pregabalin; PRM, primidone; TPM, topiramate; VPA, valproate; ZNS, zonisamide.

Astrogliosis, a hallmark of numerous neurological diseases, involves upregulation of GFAP and reflects structural and functional astrocytic alterations. As the most abundant glial cell type in the central nervous system, astrocytes regulate synaptic transmission and ion homeostasis. Together with pre‐ and postsynaptic neurons, they form the tripartite synapse and contribute to synaptogenesis, synaptic remodeling, and neurotransmitter clearance.^12^ Previous studies highlight their crucial role in epileptogenesis and seizure modulation. Astrocytic dysfunctions implicated in epilepsy include impaired potassium buffering,^13^, ^14^ altered glutamate uptake and metabolism,^15^, ^16^ mislocalization of water channels,^17^, ^18^ lipid accumulation,^19^, ^20^ astrocyte uncoupling,^21^, ^22^ and others.^23^, ^24^, ^25^, ^26^, ^27^, ^28^, ^29^ Collectively, these changes modulate neuronal excitability and epileptic activity. Accordingly, astrocytes represent a promising target for the development of novel antiepileptic therapies,^30^, ^31^ prompting increasing research interest in astrocyte function, reactivity, and heterogeneity.

Astrocytes are now recognized as a heterogeneous glial population, exhibiting diversity across brain regions and within individual brain structures.^32^, ^33^, ^34^ Recent work from our group demonstrated that astrocytes in the mouse DG, the input region of the hippocampus, can be subdivided into transcriptionally, morphologically, and physiologically distinct subtypes residing in specific DG layers.^33^ Fundamental molecular and morphological characteristics defining astrocyte heterogeneity in the murine DG appear to be conserved in the human hippocampus.^33^ These subtypes are closely interlinked with the main anatomical and functional compartments of the DG: molecular layer (ML), granule cell layer (GCL), and polymorphic layer (PL). Our data suggest that DG organization is shaped by both neuronal and astrocytic structures,^33^ making it a valuable model to study astrocyte specification in health and disease.

Based on this spatial classification,^33^ we hypothesized that DG astrocyte subtypes exhibit layer‐specific alterations under pathological conditions such as chronic epilepsy. To test this, we used DGs of patients belonging to different ILAE classes in comparison to postmortem controls (Table 1) and assessed expression levels and distribution of selected astrocytic proteins associated with structural reactivity (GFAP), altered glutamate homeostasis (the glutamate transporter EAAT2 and glutamine synthetase GS), and aberrant water regulation (water channel AQP4) in epilepsy. Extensive clinical information was available for each patient, including demographic variables, epilepsy history, pharmacological treatment, surgical outcome according to the Engel classification, and comorbidities. Thus, we provide a thorough morpho‐anatomical analysis of DG astrocyte subtypes in MTLE, contributing to a better understanding of astroglial alterations in epilepsy and their potential relevance for future astrocyte‐targeted therapies.

## 2 MATERIALS AND METHODS

### 2.1 Patient cohort

Hippocampal specimens (Table 1) were obtained from the Department of Neuropathology at the University Hospital Erlangen (approval by the Ethics‐Committee of the Friedrich‐Alexander University Erlangen‐Nürnberg, FAU: #18‐193_1‐Bio). Surgical tissue was available from patients who underwent epilepsy surgery following comprehensive presurgical evaluations, with clinical information including sex, age range at epilepsy onset, epilepsy phenotype, and MRI findings, collected from patient records and summarized in Table 1. Patients were classified according to the ILAE consensus classification of HS^10^ by experienced neuropathologists based on histopathological criteria. The cohort included 10 HS1 patients, 11 HS2 patients, and 10 noHS patients. Age‐matched postmortem hippocampal tissue from 10 individuals without epilepsy served as controls. For control cases, the postmortem interval (PMI), defined as the time between death and brain collection, ranged from 1 to 8 days (mean PMI: 3.4 days). Written informed consent for the use of resected tissue for research purposes was given by all individuals included in our study. Postsurgical seizure outcome was assessed through follow‐up interviews and documented according to the Engel classification.

### 2.2 Tissue preparation and staining of human material

Formalin‐fixed, paraffin‐embedded human hippocampal tissue was sectioned at 5 μm thickness using the Sliding Microtome HM 400R (Microm) and stored at 4°C. For deparaffinization, sections were incubated at 60°C for 1 h, followed by two 15‐min washing steps in xylene. Rehydration was performed through sequential 3‐min incubations in 100%, 95%, and 70% EtOH and subsequently, slices were washed in Milli‐Q water for 20 min. To reduce autofluorescence, slices were incubated in 0.5% NaBH_4_ (in pH 11.3 H_2_O) for 30 min. For antigen retrieval, sections were given in citric acid buffer (10 mM citric acid with 2 mM EDTA and 0.05% Tween‐20) at 95°C for 15 min, followed by a 20‐min cool‐down in the same buffer. Sections were permeabilized in PBS containing 1% TritonX‐100 for 30 min, then washed in PBS with Tween‐20 and in PBS for 2 min each. Slices were then incubated with primary antibodies diluted in blocking solution (3% NDS and 0.25% TritonX‐100) at 4°C for 72 h. After washing two times 15 min in PBS, slices were incubated overnight at 4°C with secondary antibodies diluted in blocking solution. Nuclei were stained by incubation with 4′,6‐Diamidino‐2‐phenylindole (DAPI) (1:10.000 dilution in PBS) for 10 min and slices were rinsed to remove unbound secondary antibodies by two 15‐min washing steps in PBS. Sections were then mounted using Aqua‐Poly/Mount (Polysciences, PN 18606) and stored at 4°C.

The following primary antibodies were used: chicken anti‐GFAP (1:800; Abcam, RRID: AB_304558), rabbit anti‐GFAP (1:1000; Agilent, RRID: AB_2811722), mouse anti‐GFAP (1:400; Merck, RRID: AB_477010), rabbit anti‐EAAT2 (1:400; Abcam, RRID: AB_941782), mouse anti‐EAAT2 (1:800; BD Bioscience, RRID: AB_399172), mouse anti‐EAAT1 (1:200; Miltenyi, RRID: AB_10829302), rabbit anti‐aquaporin4 (1:400; Merck, RRID: AB_258270), rabbit anti‐GS (1:400; ThermoFisher, RRID: AB_2546416) and mouse anti‐GS (1:800; ThermoFisher, RRID: AB_2735204). The following secondary antibodies were used at 1:400 dilution in blocking solution: Alexa488‐conjugated donkey anti‐mouse (ThermoFisher, RRID: AB_141607), CF488A‐conjugated donkey anti‐chicken (Biotium, RRID: AB_10854387), CF488A‐conjugated donkey anti‐rabbit (Biotium, RRID: AB_10559669), Cy3‐conjugated donkey anti‐rabbit (Jackson, RRID: AB_2307443), Cy3‐conjugated donkey anti‐mouse (Jackson, RRID: AB_2340813), Cy5‐conjugated donkey anti‐mouse (Jackson, RRID: AB_2340820) and Cy5‐conjugated donkey anti‐rabbit (Jackson, RRID: AB_2340607). As negative controls, staining was performed with secondary antibodies only (Figure S5). Fluorescence imaging was performed using the inverted fluorescence microscope Zeiss Axio Observer 7 (ApoTome.2) equipped with an Axiocam 503, a Colibri 7 LED light source and 5x, 20x, and 63x objective lenses. ZEN 3.6 software (Version 3.6.095.02000) was used for apotome acquisition. Confocal images were taken using Zeiss LSM780 with four lasers (405, 488, 550, and 633 nm) and a 63x oil immersion objective (alpha Plan‐Apochromat 63x/1.46 Oil Korr M27). Zen 2.3 SP1 FP3 software (Version 14.0.0.0) was used for confocal acquisition. Image processing and analysis were carried out using Fiji ImageJ version 1.54p and Adobe Photoshop Web.

### 2.3 Average intensity measurement of GFAP

The GFAP signal intensity was quantitatively analyzed in 10 control, 10 HS1, 10 HS2, and 9 noHS individuals. Tile‐scan images of GFAP staining were acquired using the 20x objective. GFAP expression profiles were measured using the Plot Profile tool in Fiji ImageJ, oriented from the ML to the PL. Regions of interest (ROIs) were defined as 600 μm long and 50 μm wide, with the center of the ROI in the center of the GCL. Per individual, 10–50 ROIs, evenly distributed across the whole length of the DG, were measured. The x‐axis represents the longitudinal length (600 μm) of the ROI, while the y‐axis reflects the averaged pixel intensity (API) of GFAP, measured in 0.454 μm steps. All ROIs per individual were averaged and finally data were pooled across individuals to produce an average intensity distribution curve for each cohort. No data points were excluded from the analysis.

### 2.4 Quantification and statistical analysis

Quantification of astrocyte numbers across the four groups (*n* = 7–8 per group) was performed using 20x magnification apotome images. For each individual, 3–11 images were acquired, depending on the size of the DG. ROIs were manually defined to quantify GS‐positive astrocytes. The ML was delineated from the GCL to the hippocampal fissure. A comparable sized ROI was placed in the PL, directly below the GCL. GS‐positive astrocytes were identified and counted based on GS immunoreactivity co‐localized with a DAPI‐positive nucleus. Representative images are provided in Figure S6A. For each individual, astrocytes were counted across the entire DG. Cell counts were normalized to the area measured using Fiji ImageJ version 1.54p. Individual patient data are represented as red dots in the graphs shown in Figure 2. Statistical analyses were conducted using Microsoft Excel and Graph Pad Prism version 10.0.2. Normality of data distribution was confirmed using the Shapiro–Wilk test. Accordingly, statistical comparisons were performed using one‐way ANOVA followed by a Tukey's post hoc test for multiple comparisons (Graph Pad Prism version 10.0.2). No additional corrections for multiple testing were applied. A *p*‐value <0.05 was considered statistically significant. No data points were excluded from the analysis.

### 2.5 Coefficient of variation analysis of EAAT2 and AQP4

To quantify spatial heterogeneity of staining intensity within the ML of the DG, the coefficient of variation (CV) of pixel intensity was calculated as the ratio of the standard deviation of pixel intensity to the mean pixel intensity. Tile‐scan images of EAAT2 and AQP4 staining were acquired using the 20x objective from 8 control, 5 HS1, 5 HS2, and 5 noHS cases. Five ROIs (1000 × 300 μm) per patient were evenly distributed across the ML of the DG to ensure representative sampling. CV values were first averaged per patient and subsequently averaged across each cohort. Higher CV values indicate greater variability in local signal intensity, whereas lower CV values indicate a more homogeneous signal distribution. Statistical comparisons between groups were performed using one‐way ANOVA followed by a Tukey's post hoc test for multiple comparisons (Graph Pad Prism version 10.0.2). A *p*‐value <0.05 was considered statistically significant. No data points were excluded from the analysis.

### 2.6 Relative intensity analysis of EAAT2 and AQP4

Relative intensity measurements for EAAT2 and AQP4 immunoreactivity were performed using 20x objective images from 8 control, 5 HS1, 5 HS2, and 5 noHS cases. ROIs were distributed across the entire DG and placed within the ML and the PL. ROI size was set to 1000 μm in width and 300 μm in height for the ML, and 1000 μm in width and 100 μm in height for the PL. Relative intensity values were calculated as the ratio of mean ML signal intensity to mean PL signal intensity (ML/PL ratio). A total of five ROIs per layer were analyzed for each patient and the resulting values were averaged to obtain one representative value per individual case. Subsequently, mean values were calculated for each cohort. Statistical comparisons between groups were performed using one‐way ANOVA followed by a Tukey's post hoc test for multiple comparisons (Graph Pad Prism version 10.0.2). A *p*‐value <0.05 was considered statistically significant. No data points were excluded from the analysis.

### 2.7 Manders´ Colocalization coefficient analysis between EAAT2 and AQP4

To assess spatial overlap between EAAT2 and AQP4 signals within the ML of the DG, Manders´ colocalization analysis was performed using the Coloc2 plugin in Fiji Image J version 1.54p. Tile‐scan images of EAAT2/AQP4 co‐stained sections were acquired with a 20x objective from 8 control, 5 HS1, 5 HS2, and 5 noHS cases. After background correction, five ROIs (1000 × 300 μm) per patient were evenly distributed across the ML of the DG. Threshold‐based binary masks were generated by duplicating the background‐corrected channels and applying predefined threshold values across all images. Manders´ coefficients were calculated to quantify the fraction of signal overlap between channels, with M1 representing the fraction of EAAT2 signal overlapping with AQP4 and M2 representing the fraction of AQP4 signal overlapping with EAAT2. Coefficients obtained from multiple ROIs were averaged within each patient. These values were then used for group‐level comparisons between control and epilepsy cohorts. Statistical comparisons between cohorts were performed using one‐way ANOVA followed by a Tukey's post hoc test for multiple comparisons (Graph Pad Prism version 10.0.2). A *p*‐value <0.05 was considered statistically significant. No data points were excluded from the analysis.

## 3 RESULTS

### 3.1 Astrocyte marker expression in the healthy human DG reveals pronounced layer‐specific heterogeneity

The DG was selected as our primary region of interest due to its central role in hippocampal information processing, particularly relevant in the context of MTLE.^35^, ^36^, ^37^, ^38^ In adult humans, the DG harbors approximately 11 molecularly diverse astrocyte subtypes,^33^ as identified by single‐nuclei (sn)RNA‐sequencing.^39^ Comparable to the mouse, some of these subpopulations localize to distinct DG compartments. To sharpen these initial findings, we performed a comprehensive protein‐level characterization of astrocyte subtypes in postmortem human DG tissue from control individuals. DAPI nuclear staining (Figure 1A) delineated the laminar organization of the DG, with a dense GCL and lower nuclear density in the ML and PL, providing an anatomical framework for the subsequent marker‐based analysis of astrocyte subtypes. Immunolabeling for GFAP (Figure 1B; Figure S1A) exhibited a striking layer‐specific distribution. In the PL and adjacent CA4 region, astrocytic somata and their thick primary processes were clearly detectable, whereas the ML was dominated by a meshwork of GFAP‐positive processes without visible cell bodies, which suggests a predominantly fibrous expression pattern. This layer‐specific GFAP distribution markedly diverged from the mouse DG, where GFAP is uniformly expressed across layers.^33^ GS, an astrocyte‐specific enzyme that converts glutamate to glutamine, which is then shuttled back to the presynaptic neuron, was consistently present in the cytoplasm of astrocytes across all DG layers (Figure 1C; Figure S2A), with strongest signals in ML astrocytes. The glutamate transporter EAAT2 displayed a prominent, layer‐specific expression in a subset of ML astrocytes, where it localized throughout the entire astrocyte. The expression in the PL was considerably lower with protein localization predominantly in the soma (Figure 1D; Figure S3A). This pattern likely reflects increased glutamate clearance demands in the synapse‐rich outer layers of the DG.^40^, ^41^, ^42^ EAAT1 showed a more diffuse distribution, with pronounced expression in the subgranular zone (Figure 1E). AQP4 was strongly expressed in PL astrocytes, while the signal in the ML was restricted to a subcohort of astrocytes with clear labeling throughout the entire cell (Figure 1F; Figure S4A). Overall, none of the analyzed markers was uniformly expressed across DG layers. Instead, each marker displayed pronounced layer‐specific expressions, indicating a higher degree of astrocyte layer‐specificity in the human compared with the murine DG.

**FIGURE 1:**
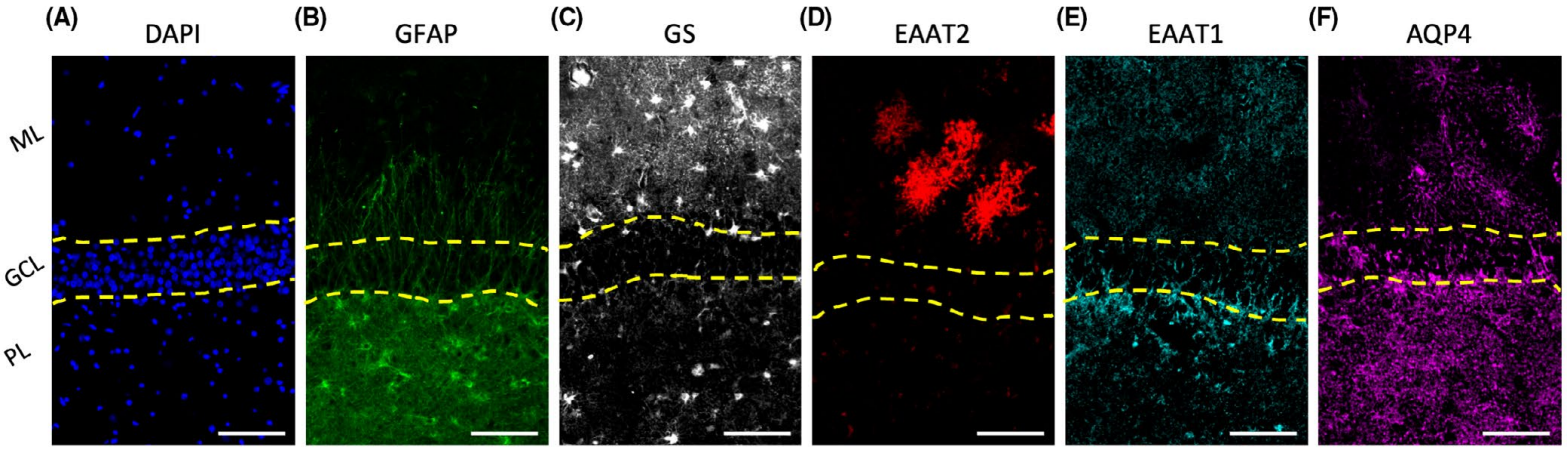
Layer‐specific expression patterns of astrocytic markers in the human dentate gyrus (DG). Representative immunofluorescence images of the DG from postmortem tissue of control (Ctrl) individuals. Staining for (A) 4′,6‐Diamidino‐2‐phenylindole (DAPI; blue), (B) glial fibrillary acidic protein (GFAP; green), (C) glutamine synthetase (GS; gray), (D) excitatory amino acid transporter 2 (EAAT2; red), (E) excitatory amino acid transporter 1 (EAAT1; cyan), and (F) aquaporin‐4 (AQP4; magenta) highlights the distinct laminar distribution of astrocytic markers across the molecular layer (ML), granule cell layer (GCL) and polymorphic layer (PL). Dashed yellow lines delineate the anatomical layers. Images captured at 20× magnification. Scale bars: 100 μm.

### 3.2 GFAP upregulation across MTLE cohorts marks reactive gliosis without a concomitant increase in astrocyte numbers

To investigate whether astrocyte heterogeneity in the control DG is preserved, altered, or accentuated under pathological conditions, we analyzed hippocampal tissue from patients with medically intractable MTLE who underwent surgical resection. MTLE with HS can be classified into subtypes based on neuronal loss and astrogliosis. In this study, the following cases were analyzed: HS Type 1 (*n* = 10), HS Type 2 (*n* = 11), and noHS cases (*n* = 10). Despite variable neuronal loss, reactive gliosis represents a consistent pathological feature in all cases and thus appears to be critically involved in the pathophysiology of MTLE. This aligns with previous evidence suggesting that noHS may constitute a distinct disease entity predominantly driven by inflammation and astrocyte dysfunction.^11^ Reactive astrogliosis encompasses diverse biochemical, functional, and morphological astrocytic alterations and is often accompanied by GFAP upregulation. All MTLE subgroups exhibited elevated GFAP immunoreactivity compared with control individuals, confirming robust reactive astrogliosis (Figure 2A–D; Figure S1A–D). However, we observed layer‐ and cohort‐specific changes in GFAP intensity and localization. In controls, GFAP expression in the PL was predominantly soma‐associated and confined to primary processes. In contrast, HS1 and HS2 patients exhibited a more peripherally distributed, fibrillar GFAP pattern with increased interlaminar distribution. This transition was particularly strong in the PL and CA4, where individual astrocytes were no longer detectable (Figure 2B,C; Figure S1B,C). Especially in HS1, a dense meshwork of GFAP‐positive fibers extended into the ML, contributing to pronounced upregulation of GFAP in this layer. The noHS group, though upregulated, retained a cellularly localized GFAP expression, allowing visualization of individual astrocytic somata (Figure 2D; Figure S1D). To compare GFAP levels across cohorts, we conducted average intensity measurements within standardized ROIs spanning from ML to PL (Figure 2E). In controls, GFAP expression in the ML showed a low baseline that steadily increased toward the PL, where it reached a plateau at a higher level (Figure 2F, blue line), underlining the layer‐specific GFAP expression as seen in Figures 1B and 2A. MTLE cohorts showed an overall trend toward upregulation, strongest in HS1 and HS2 (Figure 2F, pink and green line, respectively). The noHS cohort displayed GFAP levels in ML and GCL that largely resembled those observed in controls (Figure 2F, orange line), while all three MTLE cohorts converged to similarly elevated levels in the PL. NoHS exhibited the steepest ML‐to‐PL gradient, indicating the most pronounced layer‐specificity of GFAP distribution across DG layers. HS1 and HS2 exhibited markedly elevated GFAP signal levels in the ML, and the difference in GFAP intensity between ML and PL was less pronounced compared with the gradient observed in noHS. This was due to a stronger GFAP upregulation in the ML relative to PL, consistent with the observation that reactive astrocyte processes, especially in HS1, infiltrated the GCL and ML, blurring laminar boundaries. The analysis confirmed a layer‐specific GFAP upregulation across all MTLE cohorts compared with controls.

**FIGURE 2:**
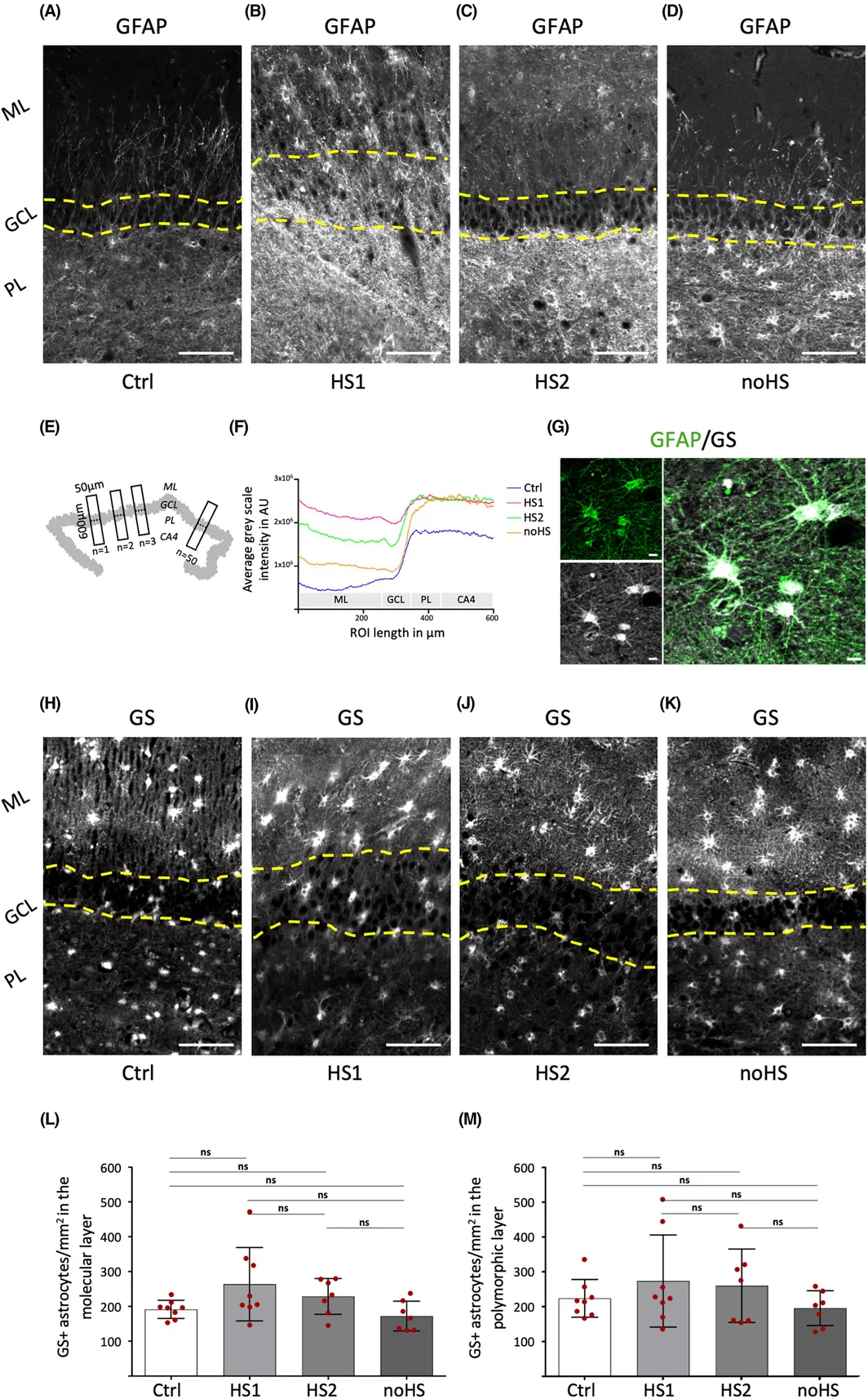
Subtype‐specific increase of GFAP expression in the DG of MTLE patients occurs independently of changes in total astrocyte numbers. (A–D) Representative GFAP immunofluorescent images from (A) Ctrl, (B) HS1, (C) HS2, and (D) noHS cases reveal altered morphology and intensity of marker expression across the DG layers and among the patient cohorts. (E) Quantification of GFAP signal intensity using standardized rectangular regions of interest (ROIs) spanning from the ML to the PL (600 × 50 μm). (F) Quantitative analysis demonstrates increased GFAP signal intensity in all MTLE cohorts across the layers of the DG in comparison to control cohort (*n* = 9–10 per group), AU = arbitrary unit. (G) Co‐immunostaining for GFAP (green) and GS (white) in control tissue shows overlapping expression in PL astrocytes. (H–K) GS expression remains consistent across all patient groups. (L), (M) Quantification of GS‐positive astrocytes in the ML and PL demonstrates no significant intergroup differences. Data are presented as mean +/− SD with individual data points (*n* = 7–8 per group). Statistical analysis: One‐way ANOVA with Tukey's post hoc test; ns = not significant. Images (A–D) and (G–K) captured at 20x magnification. Scale bars in (A–D) and (H–K): 100 μm, scale bars in (G): 10 μm.

We next asked whether reactive gliosis coincided with increased astrocyte numbers, as GFAP upregulation is often, and often wrongly, related to glial proliferation. GFAP, while widely used as a marker of astrocyte reactivity, does not permit reliable and accurate quantification of astrocyte numbers for several reasons: (i) it is difficult to identify individual astrocytes, particularly in sclerotic tissue, due to its fibrous expression; (ii) the strong upregulation of GFAP level itself is often mistaken for an upregulation of astrocyte numbers. To quantify astrocytes, we required a marker to unequivocally identify astrocytes across all DG layers and in the different MTLE classes. GS fulfilled these criteria, especially since GS was the only analyzed marker that did not change its expression level/pattern upon MTLE. Co‐immunostaining of GS with layer‐specific astrocyte markers [GFAP for PL astrocytes (Figure 2G), AQP4/EAAT2 for ML astrocytes (Figure 3A,B)] revealed that GS was expressed by most astrocytes across the DG layers. Even though GS was much stronger expressed in ML astrocytes compared with astrocytes residing in the PL, GS‐positive astrocytes could be reliably identified across DG compartments and were quantified based on the presence of clearly identifiable DAPI nuclei (Figure S6A). GS expression remained stable and cellularly localized in all MTLE cohorts (Figure 2H–K; Figure S2A–D). Furthermore, quantification revealed no significant differences in the number of GS‐positive astrocytes between MTLE cohorts and controls in the ML or PL (Figure 2L,M). These findings align with previous literature suggesting that reactive astrogliosis involves hypertrophy, cytoskeletal reorganization, and gene expression changes rather than necessarily reflecting astrocyte proliferation.^43^, ^44^ Together, layer‐specific astrocyte organization is maintained in MTLE, indicating subtype‐specific astrocyte reactivity mechanisms.

**FIGURE 3:**
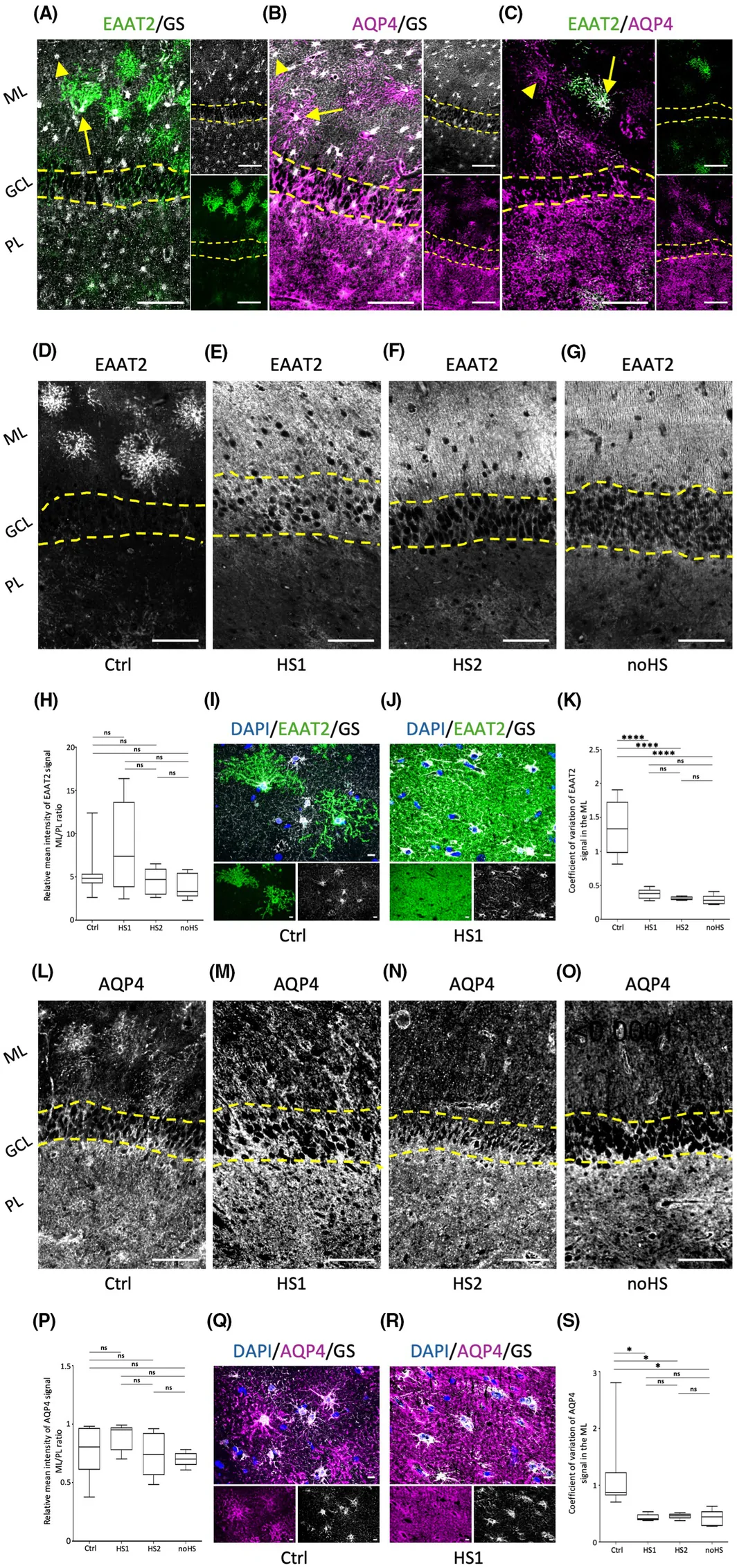
Astrocyte subtype‐specific alterations in the DG of MTLE patients. (A–C) Representative immunofluorescent images of astrocyte subpopulations in the ML of control DG tissue, identified by co‐immunostaining for EAAT2 (green), AQP4 (magenta) and GS (white). (A) GS+/EAAT2+ (arrow) and GS+/EAAT2‐ (arrowhead) astrocytes, (B) GS+/AQP4+ (arrow) and GS+/AQP4‐ (arrowhead) astrocytes, (C) EAAT2+/AQP4+ (arrow) and EAAT2‐/AQP4+ (arrowhead) astrocytes. (D–G) EAAT2 expression in the DG across patient cohorts shows altered spatial distribution in MTLE compared with controls. (H) Relative mean intensity analysis of EAAT2 expression (ML/PL ratio) demonstrating preserved layer‐specific enrichment of EAAT2 in the ML across cohorts. (I), (J) High‐magnification images demonstrate reorganization of EAAT2 (green) expression in the ML of a representative HS1 patient compared with a control case despite preserved GS (white) immunoreactivity, DAPI in blue. (K) Coefficient of variation (CV) analysis of EAAT2 signal distribution in the ML. (L–O) AQP4 expression shows reorganization in MTLE patients compared with controls across the layers of the DG. (P) Relative mean intensity analysis of AQP4 expression (ML/PL ratio) demonstrating preserved layer‐specific enrichment of AQP4 in the PL across cohorts. (Q), (R) High‐magnification images from the ML demonstrate reorganization of AQP4 (magenta) expression of a representative HS1 patient compared with a control case despite preserved GS (white) immunoreactivity, DAPI in blue. (S) CV analysis of AQP4 signal distribution in the ML. Data in (H), (K), (P), and (S) are presented as box plots showing median, interquartile range (box) and 5th‐95th percentiles (whiskers) (*n* = 5–8 per group). Statistical analysis: One‐way ANOVA with Tukey's post hoc test; ns = not significant; * *p* < 0.05, **p < 0.01, ***p < 0.001, ****p < 0.0001. Images (A–G) and (L–O) captured at 20x magnification. Images (I), (J), (Q), and (R) captured at 63x magnification. Scale bars in (A–G) and (L–O): 100 μm, scale bars in (I), (J), (Q), and (R): 10 μm.

### 3.3 Astrocyte subtype‐specific alterations of EAAT2 and AQP4 in the DG of MTLE patients

To capture alterations of DG astrocyte subtypes in MTLE, we analyzed EAAT2 and AQP4 expression. AQP4 and EAAT2 were strongly expressed in individual astrocytes in the ML of postmortem controls, reflecting distinct astrocyte subgroups within the ML. Specifically, we detected GS+/EAAT2+ (Figure 3A, arrow), GS+/EAAT2‐ (Figure 3A, arrowhead), GS+/AQP4+ (Figure 3B, arrow), GS+/AQP4‐ (Figure 3B, arrowhead), EAAT2+/AQP4+ (Figure 3C, arrow), and EAAT2‐/AQP4+ (Figure 3C, arrowhead) astrocyte subtypes. This diversity reveals a remarkable degree of astrocyte heterogeneity in the human DG, even within a single compartment, reflecting functional specialization through differential expression of membrane transport and channel proteins. In MTLE, subtype‐specific expression patterns of astrocytic markers were profoundly disrupted. Across all MTLE cohorts, EAAT2 immunoreactivity appeared markedly diffuse and less structured compared with control individuals, particularly within the ML (Figure 3D–G; Figure S3A–D). Relative mean intensity analysis showing the ML/PL ratio demonstrated that the characteristic layer‐specific enrichment of EAAT2 remained preserved across all cohorts despite the altered cellular appearance, with a consistent predominance of EAAT2 signal in the ML across all groups (Figure 3H). The well‐defined, membrane‐associated EAAT2 signal outlining individual astrocytes in controls (Figure 3I) was replaced by a homogeneous signal distribution in the ML of MTLE patients, obscuring individual astrocytic boundaries (Figure 3J). This blurred expression of EAAT2 was ML‐specific and did not spread to other DG compartments, indicating a highly subtype‐specific phenotype. To further quantify this redistribution, we determined the CV of EAAT2 signal within the ML as the ratio of signal standard deviation to mean intensity. Compared with controls, all MTLE cohorts showed significantly reduced CV values (Figure 3K), quantitatively confirming the loss of spatially restricted EAAT2 localization and the transition toward a diffuse and more homogeneous signal pattern. This phenotype was recapitulated by AQP4, which is expressed in most EAAT2+ ML astrocytes (Figure 3C). While in control tissue, AQP4 delineated individual ML astrocytes (Figure 3L; Figure S4A), AQP4 labeling in MTLE patients appeared diffusely distributed without discernible cellular boundaries (Figure 3M–O; Figure S4B–D). Relative mean intensity analysis of the ML/PL ratio revealed preservation of the characteristic layer‐specific expression profile of AQP4 across all cohorts (Figure 3P), with preferential enrichment in the PL. Representative higher magnification images further illustrate the loss of clearly discernible membrane‐associated AQP4 localization in MTLE tissue compared with controls (Figure 3Q,R). Quantitative assessment of signal heterogeneity demonstrated significantly reduced CV values in MTLE tissue compared with controls (Figure 3S), consistent with a more homogeneous and widespread distribution of AQP4 labeling throughout the ML. Preserved GS immunoreactivity across cohorts further indicated the persistence of ML EAAT2+, AQP4+ astrocytes in MTLE despite the altered cellular localization of both proteins (Figure 3I,J,Q,R). To further characterize the spatial relationship between EAAT2 and AQP4, we quantified their overlap using Manders' colocalization coefficients. M1 represents the fraction of EAAT2 signal overlapping with AQP4, whereas M2 represents the fraction of AQP4 signal overlapping with EAAT2. In control tissue, M1 values were significantly higher than M2 values, indicating that EAAT2 signal largely overlapped with AQP4, whereas AQP4 exhibited a broader distribution extending beyond EAAT2‐positive regions (Figure S6B,C). This observation is consistent with the coexistence of EAAT2+/AQP4+ and EAAT2‐/AQP4+ astrocyte populations observed in Figure 3C. Across MTLE cohorts, M1 and M2 values converged and displayed comparable degrees of reciprocal overlap between EAAT2 and AQP4 (Figure S6B,C), which is supported by representative images illustrating the altered EAAT2/AQP4 distribution pattern across cohorts (Figure S6D). These findings indicate a loss of the physiological asymmetric overlap pattern between EAAT2 and AQP4, characterized by the extensive overlap of EAAT2 signal with AQP4 signal and the broader distribution of AQP4 signal beyond EAAT2‐positive regions, and suggest a converging spatial redistribution of both proteins within the ML of MTLE patients.

Here, we demonstrate that pathological astrocyte reactivity in MTLE is not a uniform process but comprises complex, subtype‐specific alterations that extend beyond the classical upregulation of GFAP. Importantly, astrocyte subtypes retain their layer‐specific molecular profile even upon severe forms of MTLE. The aberrant cellular and noncellular redistribution and mislocalization of functionally relevant astrocyte proteins suggest a dysregulation of crucial functions, including glutamate and water homeostasis.

## 4 DISCUSSION

Our data provide evidence that astrocyte heterogeneity, previously described in the healthy murine and human DG,^33^ is largely preserved under pathophysiological conditions in MTLE, albeit with distinct region‐ and subtype‐specific alterations in marker expression. Analysis of healthy human DG tissue revealed that, unlike in the mouse brain, not a single marker was uniformly expressed in DG astrocytes, indicating a more pronounced astrocyte diversity in humans. Evolutionary, higher number of glial cells is concomitant with higher brain function. In simpler organisms such as *Drosophila melanogaster* and *Caenorhabditis elegans*, the neuron‐to‐glia ratio is strongly shifted toward neurons,^45^, ^46^, ^47^ while in mammalians it is balanced. The spatial diversification of astrocytes in the human DG suggests that both astrocyte number and specialization may have evolved to support cognition and executive functions.

Astrocyte involvement in epilepsy is well established and reactive gliosis characterized by GFAP upregulation is a fixed diagnostic parameter.^10^ However, reactive gliosis is interpreted differently across studies, and research in this field suffers from this lack of mutual understanding.^43^, ^48^, ^49^, ^50^, ^51^, ^52^ While reactive gliosis involves morphological, molecular, and functional remodeling of astrocytes in response to disease or injury, it is often erroneously equated with the generation of new astrocytes. Astrogenesis mainly occurs after acute CNS injury to form glial scars. This process is largely confined to the early post‐injury phase,^44^, ^53^ and is very rare in chronic diseases. While a recent study^54^ reported ongoing gliogenesis and presence of immature astroglia in MTLE, we found no differences in total astrocyte numbers, consistent with the chronic nature of epilepsy.

Astrocyte reactivity in epilepsy extends beyond GFAP upregulation and includes alterations in glutamate and water transport (EAAT2 and AQP4, respectively) as implicated already by other studies.^55^, ^56^, ^57^ AQP4 modulates neuronal excitability and seizure susceptibility through water and ion homeostasis,^58^, ^59^, ^60^ and impaired EAAT2 expression may cause glutamate accumulation and excitotoxicity.^61^, ^62^, ^63^ Beyond altered expression levels, we observed EAAT2 and AQP4 mislocalization toward uniform, non‐cell‐specific patterns indicative of a loss‐of‐function. This aberrant expression occurred in all ML astrocyte subtypes, independent of the MTLE class, potentially reflecting a shared reactive trajectory. Such spatially nuanced alterations imply a highly localized process in MTLE affecting specific DG layers and associated astrocyte subtypes, rather than a global gliotic transformation uniformly involving all astrocytes. These findings refine previous studies demonstrating that astrocyte identity is not only developmentally imprinted but also shaped by local neuronal activity and tissue architecture.^64^, ^65^, ^66^

Our study combined high‐resolution immunofluorescent imaging of canonical astrocytic markers with the international HS classification^10^ and a validated spatial characterization of astrocyte subtypes,^33^ allowing a robust analysis of subtype‐specific changes in MTLE. Limitations include reliance on postmortem control tissue, which, although representing the best available option, may differ from surgically resected MTLE specimens with respect to postmortem delay, tissue processing, and cellular integrity, thereby limiting direct comparability between groups. Furthermore, we acknowledge the cause‐and‐consequence dilemma, so that causal relationships between astrocytic alterations and epileptic activity remain speculative.

We propose that MTLE involves not only neuronal network dysfunction but also altered astrocyte subtype function. Whether these changes represent a regression to a less differentiated state, a downregulation of astrocyte subtype‐specific properties, or an adaptive response to chronic seizures – and whether they precede, accompany, or result from epileptic activity – remains a challenging question. Future studies are necessary to explore the functional consequences of astrocyte subtype‐specific reorganization and to answer key questions: What are the underlying mechanisms driving the diffuse redistribution of astrocyte marker expression toward the periphery in MTLE? Does the redistribution of astrocyte marker expression represent a maladaptive response to chronic seizure activity, or could it reflect compensatory mechanisms? What is the role of neuron–astrocyte interactions in initiating or maintaining these changes? Integrating our spatial findings into functional analysis, ideally combined with electrophysiological, transcriptomic, and metabolic experiments, will be a milestone in understanding astrocyte subtype‐specific alterations in MTLE and in developing novel therapeutic targets and strategies for treating epilepsy.

## 5 CONCLUSION

Astrocyte subtype diversity is a key feature of the human DG, both in physiological and pathological conditions. DG astrocytes do not respond uniformly to MTLE but undergo subtype‐specific changes in expression levels as well as localization of functionally relevant astrocytic proteins. These insights emphasize astrocyte subtype‐specific alterations as a central component of MTLE pathophysiology and may open new avenues for precision therapies that move beyond the neuron‐centered view of epilepsy by incorporating the diversity of glial cells.

## AUTHOR CONTRIBUTIONS

Conceptualization C.L., J.J.S., R.B.; Investigation C.L., J.J.S.; Formal analysis C.L., J.J.S. R.C.; Resources and Funding acquisition D.D., I.B., R.B.; Writing original draft C.L., R.B.; Supervision R.B. All authors read and approved the manuscript.

## FUNDING INFORMATION

This work was supported by grants from the German Research Foundation (DFG; BE5136/6‐1 and BE5136/7‐1 to R.B., INST 410/45‐1 FUGG). C.L. was supported by the Interdisciplinary Centre for Clinical Research (IZKF), Erlangen. The research training group 2162 “Neurodevelopment and Vulnerability of the Central Nervous System” of the DFG (GRK2162) supported J.J.S as a fellow. IB was supported by the Deutsche Forschungsgemeinschaft (DFG, German Research Foundation) project number 460333672–CRC1540 Exploring Brain Mechanics.

## CONFLICT OF INTEREST STATEMENT

All authors declare that there are no personal, professional, or financial relationships that could be potentially interpreted as a conflict of interest in relation to the submitted work.

## ETHICS APPROVAL

Hippocampal specimens were obtained from the Department of Neuropathology at the University Hospital Erlangen (approval by the Ethics‐Committee of the Friedrich‐Alexander University Erlangen‐Nürnberg, FAU: #18‐193_1‐Bio). Informed consent for the use of resected tissue for research purposes was given by all individuals included in our study. We confirm that we have read the Journal's position on issues involved in ethical publication and affirm that this report is consistent with those guidelines.

## CONSENT

All authors have approved the manuscript and agree with its submission.

## Supporting information


**Figure S1.** GFAP expression reveals altered morphology and intensity across the layers of the DG among patient cohorts.
(A) GFAP expression in representative Ctrl individuals. (B) GFAP expression in representative HS1 individuals. (C) GFAP expression in representative HS2 individuals. (D) GFAP expression in representative noHS individuals. Images captured at 20× magnification. Scale bars: 100 μm.

**Figure S2.** GS expression remains consistent across patient cohorts.
(A) GS expression in representative Ctrl individuals. (B) GS expression in representative HS1 individuals. (C) GS expression in representative HS2 individuals. (D) GS expression in representative noHS individuals. Images captured at 20× magnification. Scale bars: 100 μm.

**Figure S3.** EAAT2 expression in the DG across patient cohorts shows altered spatial distribution in MTLE compared with control DG tissue.
(A) EAAT2 expression in representative Ctrl individuals. (B) EAAT2 expression in representative HS1 individuals. (C) EAAT2 expression in representative HS2 individuals. (D) EAAT2 expression in representative noHS individuals. Images captured at 20× magnification. Scale bars: 100 μm.

**Figure S4.** AQP4 expression shows reorganization across the layers of the DG in MTLE patients compared with controls.
(A) AQP4 expression in representative Ctrl individuals. (B) AQP4 expression in representative HS1 individuals. (C) AQP4 expression in representative HS2 individuals. (D) AQP4 expression in representative noHS individuals. Images captured at 20× magnification. Scale bars: 100 μm.

**Figure S5.** Negative controls for immunofluorescence staining.
(A) Representative negative control for GFAP. (B) Representative negative control for GS. (C) Representative negative control for EAAT2. (D) Representative negative control for EAAT1. (E) Representative negative control for AQP4. Images captured at 20× magnification. Scale bars: 100 μm.

**Figure S6.** Representative GS immunoreactivity in the DG layers of a control and MTLE patient (A) and colocalization analysis of EAAT2 and AQP4 expression in the ML (B)‐(D).
(A) Representative GS immunostaining (white) with DAPI nuclear counterstaining (blue) in the DG of a control subject and MTLE patient. Higher magnification images show GS‐positive astrocytes in the ML and PL. (B) Manders´ overlap coefficient M1 representing the fraction of EAAT2 signal overlapping with AQP4 in the ML across control and MTLE patient cohorts. (C) Manders´ overlap coefficient M2 representing the fraction of AQP4 signal overlapping with EAAT2 in the ML across control and MTLE patient cohorts. (D) Representative immunofluorescence images of EAAT2 (green) and AQP4 (magenta) co‐staining in the ML across patient cohorts illustrating signal overlap patterns. Data in (B) and (C) are presented as box plots showing median, interquartile range (box), and 5th–95th percentiles (whiskers) (*n* = 5–8 per group). Statistical analysis: one‐way ANOVA with Tukey's post hoc test; ns = not significant; *p < 0.05, **p < 0.01, ***p < 0.001, ****p < 0.0001. Images in (D) captured at 20× magnification. Scale bars: 100 μm in overview images (A); 10 μm in high‐magnification images (A) and panel (D).

## Data Availability

All data generated or analyzed during this study are included in this published article and its supplementary information files.
