# Supplementary figures and images for: Astrocyte subtype‐specific alterations in the dentate gyrus of individuals with mesial temporal lobe epilepsy
Source: Epilepsia Open. 2026 Aug 1:10.1002/epi4.70324. Online ahead of print. doi: 10.1002/epi4.70324 (PMC13428351; doi:10.1002/epi4.70324)

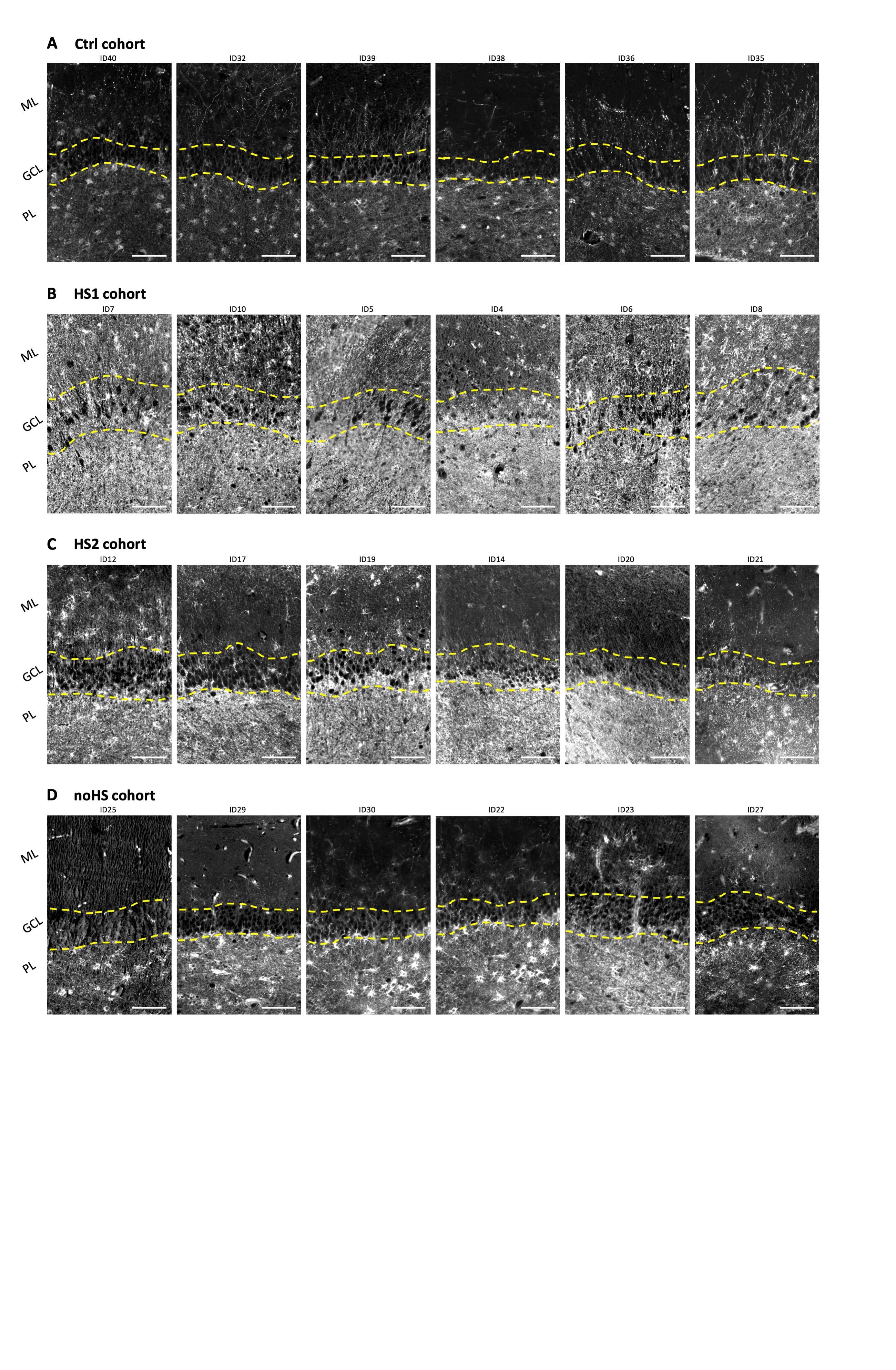

Supplement: Supplementary file 1 — Figure S1. GFAP expression reveals altered morphology and intensity across the layers of the DG among patient cohorts. (A) GFAP expression in representative Ctrl individuals. (B) GFAP expression in representative HS1 individuals. (C) GFAP expression in representative HS2 individuals. (D) GFAP expression in representative noHS individuals. Images captured at 20× magnification. Scale bars: 100 μm. [file EPI4-9999-0-s005.tiff]

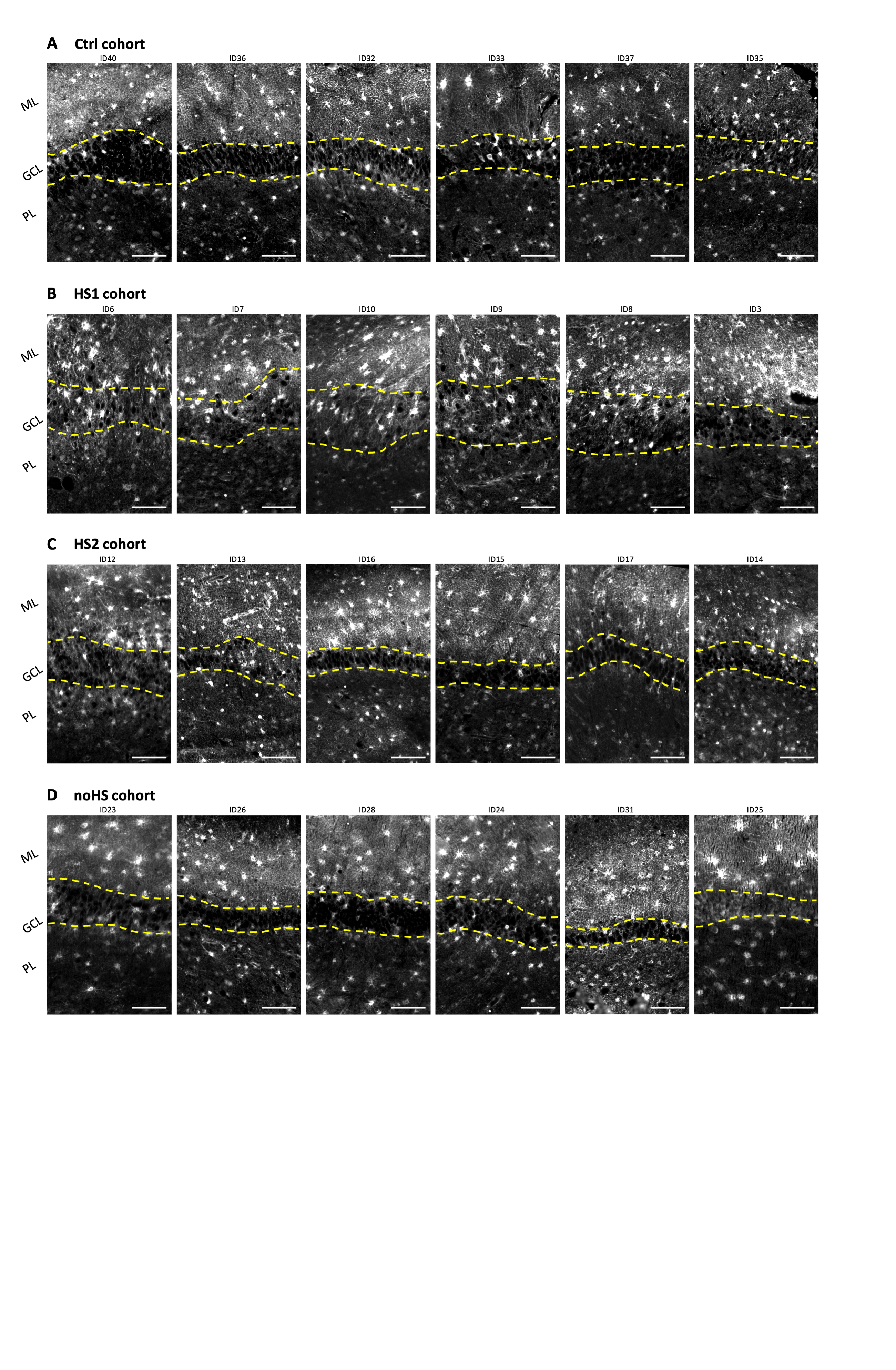

Supplement: Supplementary file 2 — Figure S2. GS expression remains consistent across patient cohorts. (A) GS expression in representative Ctrl individuals. (B) GS expression in representative HS1 individuals. (C) GS expression in representative HS2 individuals. (D) GS expression in representative noHS individuals. Images captured at 20× magnification. Scale bars: 100 μm. [file EPI4-9999-0-s006.tiff]

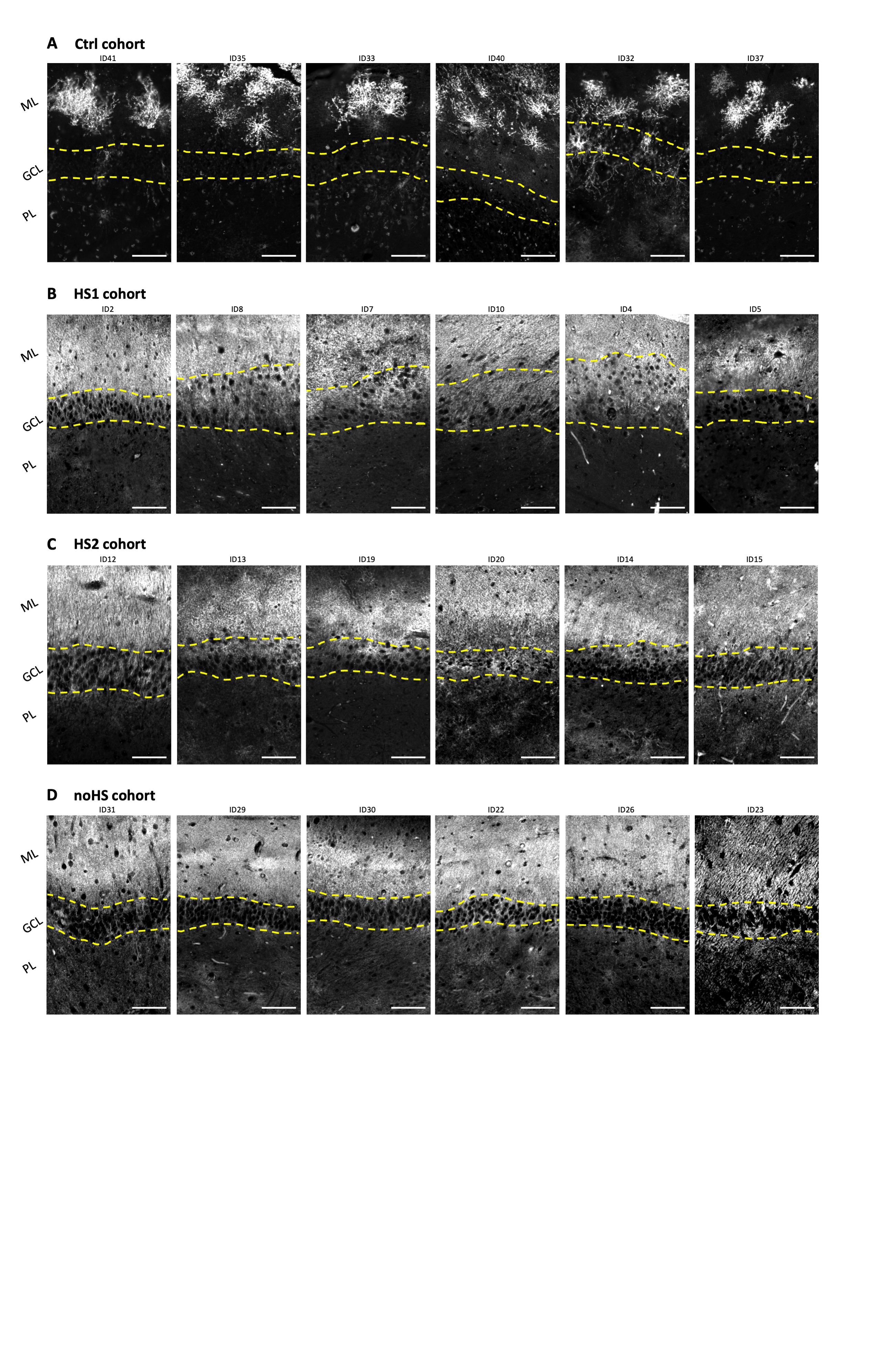

Supplement: Supplementary file 3 — Figure S3. EAAT2 expression in the DG across patient cohorts shows altered spatial distribution in MTLE compared with control DG tissue. (A) EAAT2 expression in representative Ctrl individuals. (B) EAAT2 expression in representative HS1 individuals. (C) EAAT2 expression in representative HS2 individuals. (D) EAAT2 expression in representative noHS individuals. Images captured at 20× magnification. Scale bars: 100 μm. [file EPI4-9999-0-s001.tiff]

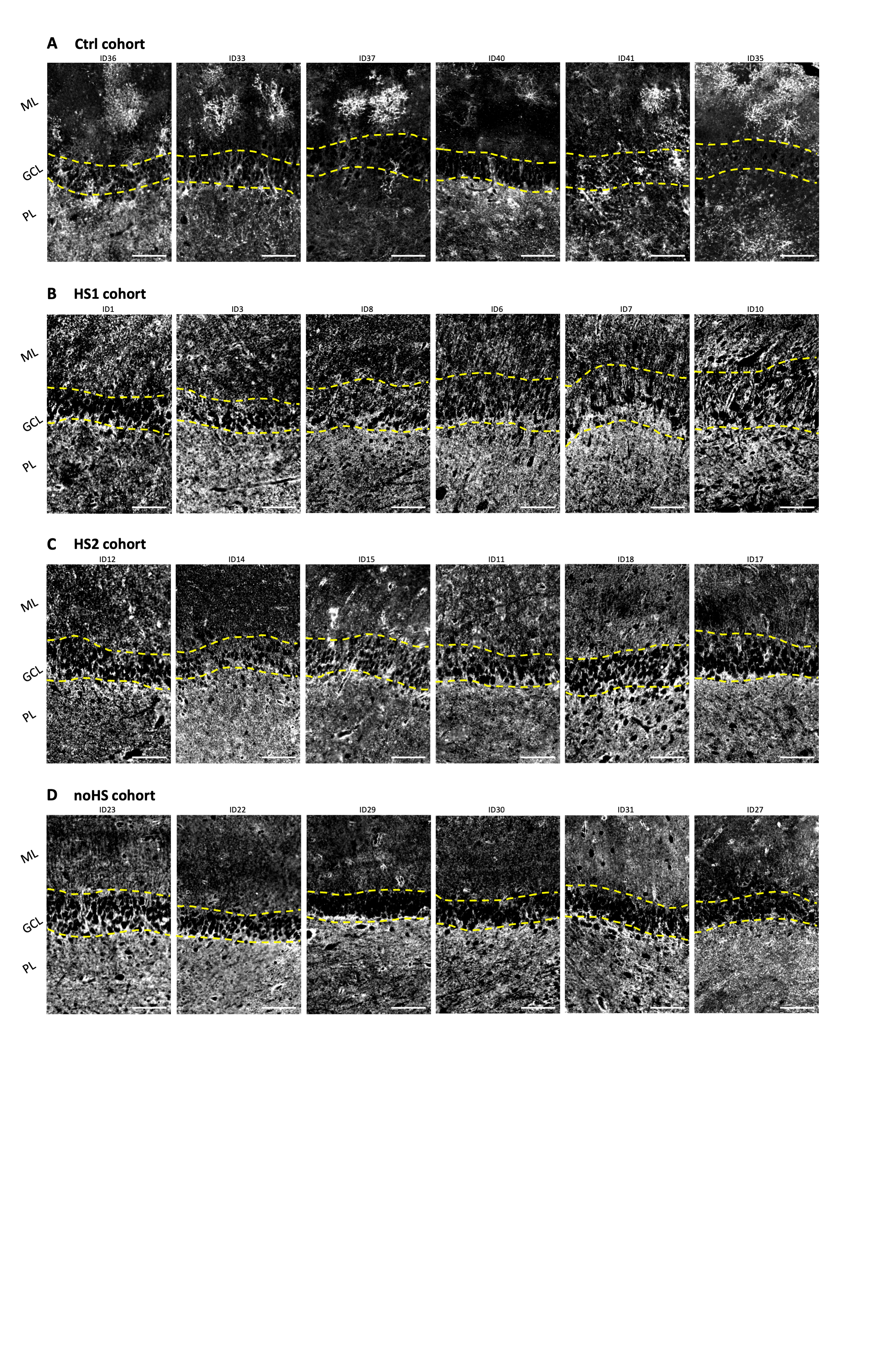

Supplement: Supplementary file 4 — Figure S4. AQP4 expression shows reorganization across the layers of the DG in MTLE patients compared with controls. (A) AQP4 expression in representative Ctrl individuals. (B) AQP4 expression in representative HS1 individuals. (C) AQP4 expression in representative HS2 individuals. (D) AQP4 expression in representative noHS individuals. Images captured at 20× magnification. Scale bars: 100 μm. [file EPI4-9999-0-s003.tiff]

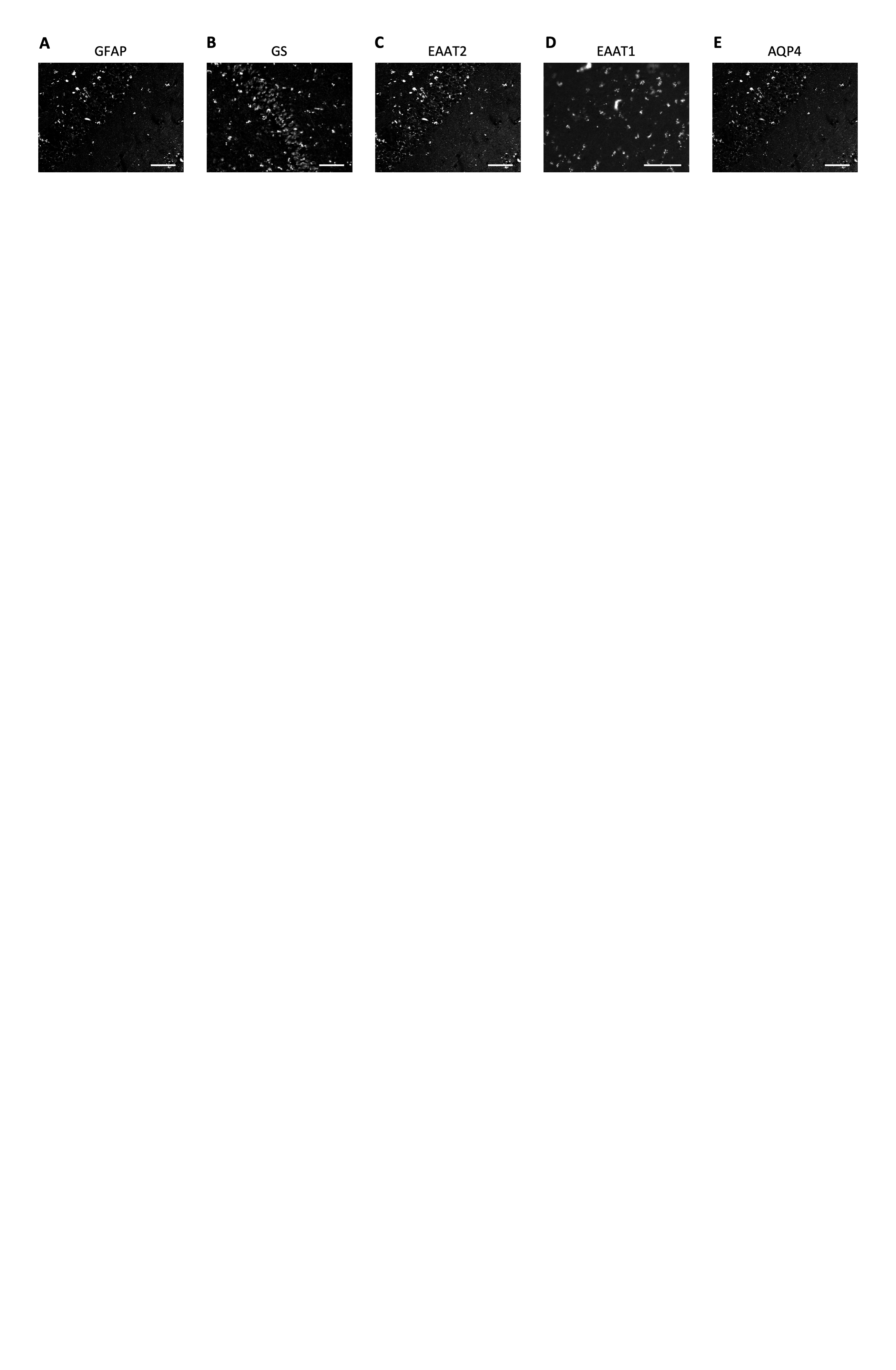

Supplement: Supplementary file 5 — Figure S5. Negative controls for immunofluorescence staining. (A) Representative negative control for GFAP. (B) Representative negative control for GS. (C) Representative negative control for EAAT2. (D) Representative negative control for EAAT1. (E) Representative negative control for AQP4. Images captured at 20× magnification. Scale bars: 100 μm. [file EPI4-9999-0-s004.tiff]

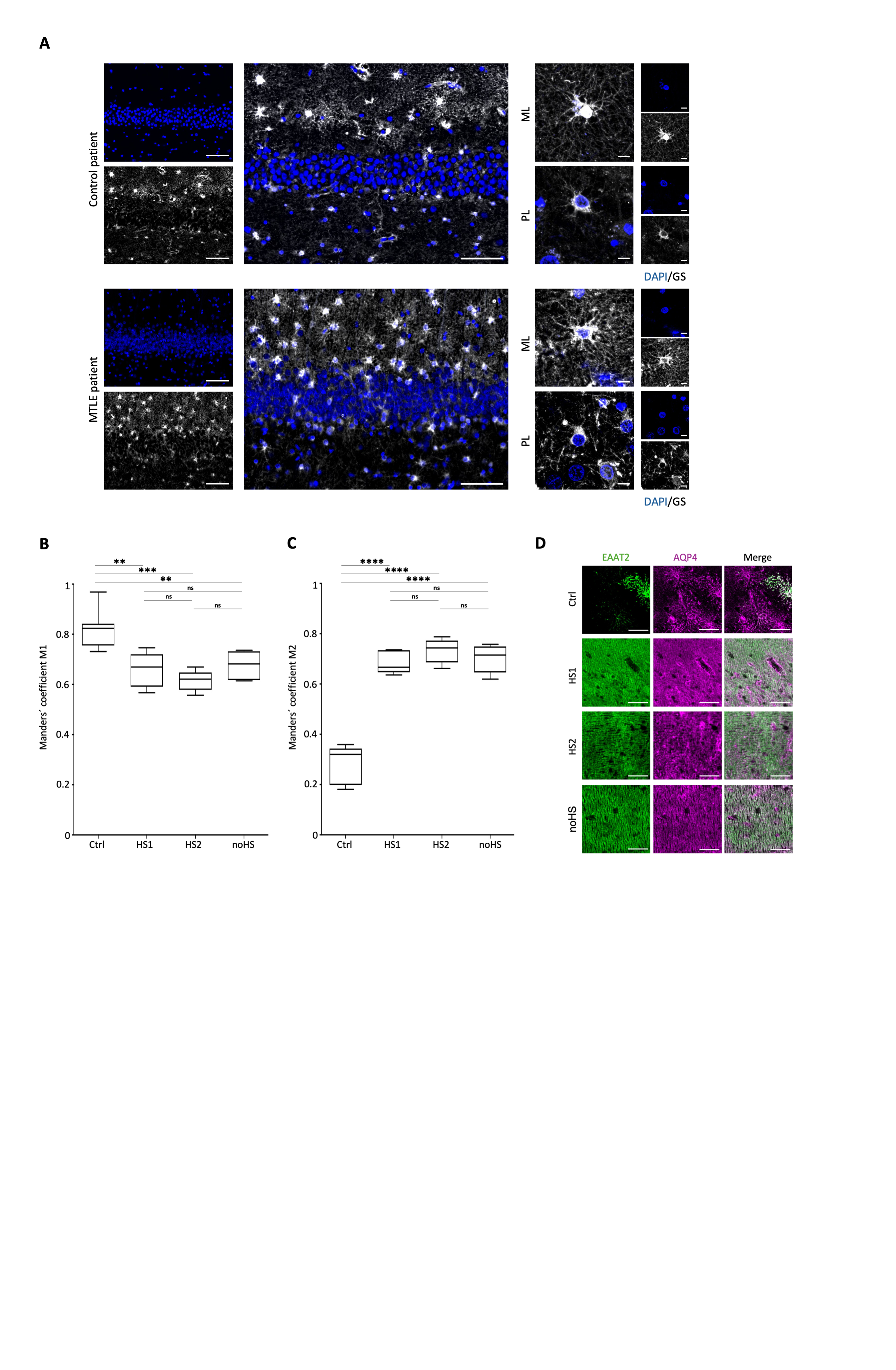

Supplement: Supplementary file 6 — Figure S6. Representative GS immunoreactivity in the DG layers of a control and MTLE patient (A) and colocalization analysis of EAAT2 and AQP4 expression in the ML (B)‐(D). (A) Representative GS immunostaining (white) with DAPI nuclear counterstaining (blue) in the DG of a control subject and MTLE patient. Higher magnification images show GS‐positive astrocytes in the ML and PL. (B) Manders´ overlap coefficient M1 representing the fraction of EAAT2 signal overlapping with AQP4 in the ML across control and MTLE patient cohorts. (C) Manders´ overlap coefficient M2 representing the fraction of AQP4 signal overlapping with EAAT2 in the ML across control and MTLE patient cohorts. (D) Representative immunofluorescence images of EAAT2 (green) and AQP4 (magenta) co‐staining in the ML across patient cohorts illustrating signal overlap patterns. Data in (B) and (C) are presented as box plots showing median, interquartile range (box), and 5th–95th percentiles (whiskers) (n = 5–8 per group). Statistical analysis: one‐way ANOVA with Tukey's post hoc test; ns = not significant; *p < 0.05, **p < 0.01, ***p < 0.001, ****p < 0.0001. Images in (D) captured at 20× magnification. Scale bars: 100 μm in overview images (A); 10 μm in high‐magnification images (A) and panel (D). [file EPI4-9999-0-s002.tiff]
